# Agricultural constraints on microbial resource use and niche breadth in drainage ditches

**DOI:** 10.7717/peerj.4175

**Published:** 2017-12-22

**Authors:** Ellard R. Hunting, S. Henrik Barmentlo, Maarten Schrama, Peter M. van Bodegom, Yujia Zhai, Martina G. Vijver

**Affiliations:** 1 Institute of Environmental Sciences, Leiden University, Leiden, Netherlands; 2 NIOO-KNAW, Wageningen, The Netherlands

**Keywords:** Biodiversity, Bacteria, Decomposition, Microorganisms, Organic matter, Ecosystem functioning

## Abstract

**Background:**

Microorganisms govern important ecosystems processes, in particular the degradation of organic matter (OM). However, microorganisms are rarely considered in efforts to monitor ecosystem health and functioning. Evidence suggests that environmental perturbations can adversely affect microbial communities and their ability to use available substrates. However, whether impacted microbial efficiencies in extracting and utilizing the available resources (resource niche breadth) translate to changes in OM degradation in natural systems remains poorly understood.

**Methods:**

Here we evaluated effects of differences in OM related to agricultural land use (OM derived from ditches adjacent to grasslands, bulb fields and a pristine dune area) on microbial functioning. We specifically assessed (1) resource niche breadths of microbial communities during initial community assembly in laboratory microcosms and already established natural communities, and (2) how changes in community resource niche breadth translates to the degradation of natural OM.

**Results:**

A disparity existed between microbial resource niche breadth in laboratory incubations and natural microbial communities. Resource utilization and niche breadth of natural microbial communities was observed to be constrained in drainage ditches adjacent to agricultural fields. This outcome coincides with retarded degradation of natural OM collected from ditches adjacent to hyacinth bulb fields. Microbial communities in bulb field ditches further showed functional redundancy when offered grassland OM of seemingly higher substrate quality.

**Discussion:**

Results presented in this study suggest that agricultural practices can impose constraints on microbial functional diversity by reducing OM resource quality, which can subsequently translate to confined microbial resource niche differentiation and reduced organic matter degradation rates. This hints that assessments of actual microbial resource utilization and niche differentiation could potentially be used to assess the ecological health and functioning of natural communities.

## Introduction

Microorganisms such as bacteria and fungi are a diverse and abundant component in soils and sediments and essential in governing the degradation of dead organic matter (OM) (e.g., Van der Heijden, Bardgett & Van Straalen, 2008; Gessner et al., 2010). Despite their importance, microorganisms are mostly absent from predictive models of biodiversity and ecosystem functioning or merely captured in bulk parameters (e.g., bacterial biomass) (cf. Sarmento et al., 2010; Treseder et al., 2012), nor are microorganisms included in current monitoring efforts aiming to assess the health and performance of ecosystems (e.g., Lau et al., 2015). However, increasing evidence suggests that microbial communities can respond to environmental perturbations (e.g., Hartman et al., 2008; Lear & Lewis, 2009; Wang et al., 2011), warranting the need to assess the effects of anthropogenic stressors on microbial communities and potential effects on ecosystem functioning.

Whether anthropogenic pressures on natural microbial communities ultimately have functional consequences on ecologically relevant scales remains speculative. Microbial decomposers, and bacteria in particular, have been viewed as functionally redundant because of a high degree of similarity among bacterial species (e.g., Langenheder, Lindström & Tranvik, 2005; Jiang, 2007; Gamfeldt, Hillebrand & Jonsson, 2008; Bell et al., 2009 and references therein). Therefore, ecosystem processes like decomposition are expected to proceed independently from microbial diversity. More recently, studies using laboratory incubations have demonstrated that ecosystem processes can benefit from higher levels of microbial species richness (Strickland et al., 2009; Peter, Beier & Bertilsson, 2011), for instance by enhancing respiration (Bell et al., 2005), increasing stability of microbial metabolism of ecosystem processes (Girvan et al., 2005; Dang, Chauvet & Gessner, 2005; Hunting et al., 2015), or providing resilience to environmental perturbations (e.g., Griffiths et al., 2000; Girvan et al., 2005; Hartman et al., 2008; Wittebolle et al., 2009). An increasing number of studies aimed to assess effects of environmental perturbations on natural microbial communities, yielding contradictive outcomes. Shifts in microbial community structure have indeed been observed to affect ecosystem processes (Strickland et al., 2009; Powell, Welsh & Hallin, 2015), but hitherto focused mainly on specific microbial processes such as denitrification. Since the majority of these studies capture effects only during the early stages of microbial community assembly in laboratory settings or specific isolated processes, the significance of anthropogenic pressures on overall measures of ecosystem functioning (i.e., carbon turnover and OM degradation) in natural settings remains uncertain.

Most studies use traditional (functional) diversity measures (e.g., species richness, metabolic diversity) to assess the functional consequences of biodiversity loss and environmental perturbations. However, functional consequences more likely result from differences in the efficiencies of microbial communities to extract and utilize the available resources, as visible in the resource niche breadth of the community (Leibold, 1995). The composition and availability of OM resources can, in turn, strongly influence microbial production and diversity (Judd, Crump & Kling, 2006; Langenheder & Prosser, 2008; Hunting et al., 2013), suggesting a complex interplay between OM diversity and quality and microbial diversity ultimately influencing the spatio-temporal properties of ecosystem processes. The concept of resource niche breadth recently gained rejuvenated emphasis in microbial ecology (Salles et al., 2009). Although increasing evidence based mostly on controlled manipulative experiments hints this approach could provide valuable information (Salles et al., 2009; Hunting et al., 2015), it has not been considered in relation to anthropogenic pressures and varying OM sources, and how this reflects the functioning of natural microbial communities.

Agricultural drainage ditches provide a valuable system to study the relative importance of anthropogenic stressors on microbial resource niche utilization and niche breadth on ecologically relevant scales as they provide a network of water bodies that harbor large differences in organic matter quality inputs and agricultural land use in close vicinity of each other, but otherwise similar environmental conditions. These microbial communities are continuously exposed to dynamic hydrological conditions and the run-off of pesticides and nutrients from adjacent agricultural fields that affect both the resident organisms and the quality of the available OM (e.g., Herzon & Helenius, 2008; Hunting et al., 2016). Hence, it is conceivable that agricultural practices and their effects on organic matter quality can either impose shifts in microbial communities or drive microbial community resource niche differentiation in drainage ditches that can potentially translate in altered ecosystem processes, in particular the degradation of organic matter (OM). Therefore, we evaluate in the present study (1) the effect of agricultural land use on the resource niche breadth and functional composition of microbial communities both during initial community assembly and under environmentally relevant stress settings, and (2) how changes in community resource niche breadth translate to OM degradation. To this end, microbial resource usage was assessed during the initial stages of community assembly in laboratory incubations, while microbial resource niche breadth and OM degradation were determined in ditches adjacent to pristine dune areas as well as intensively used grass and bulb fields.

## Methods

### DECOTAB preparation

Approaches to study OM decomposition often make use of conventional methods such as litter bags and leaf disks. However, these methods carry methodological constraints when one tries to manipulate, standardize and quantify organic matter in natural systems. Instead, we used the recently developed DEcomposition and COnsumption TABlets, DECOTABs (Kampfraath et al., 2012: http://www.decotab.org), that embed a homogenized and standardized mixture of particulate OM (POM), as an opportunity to overcome these constraints. DECOTABs were prepared following the procedures as described by Kampfraath et al. (2012) and Hunting et al. (2016). To this end, sediment containing particulate OM was collected from sediments of three different ditches adjacent to either grasslands, hyacinth bulb fields, and a pristine dune area. Re-suspension and subsequent separation by differences in weight provided sediment-free POM which was dried for seven days at 45 °C. After drying, OM was sieved (500 µm–2 mm) to discard coarse material and obtain POM that was large enough to be retained within an agar matrix. DECOTABs for both land use practices (pasture POM and hyacinth POM; hereafter called DECOTAB treatment) were prepared from 60 g/L POM and 20 g/L purified agar. The agar was dissolved in demineralized water and heated up to 100 °C. After cooling (<50 °C), POM was mixed in the solution and then poured into a polycarbonate mold (diameter: 35 mm; height: 7 mm; total volume: 770 mm^3^) to solidify at a temperature of 7 °C. Prior to further experimentation, a number of DECOTABs of each specific DECOTAB treatment were placed in deionized water for two weeks to evaluate whether DECOTABs had a proper consistency and to visually confirm that DECOTABs were not leaching OM. DECOTABs were subsequently used for the laboratory and field incubations as described below.

### Laboratory incubation: effects of organic matter treatment on microbial community assembly

DECOTAB treatments were ethanol-sterilized and placed in polyethylene microcosms (350 mL) containing dH_2_O to which a 15 mL microbial inoculum was added consisting of a 1:1 mixture of water collected from nearby ditches. Biofilms were allowed to develop for 3 weeks, and carbon substrate utilization of the developed microbial biofilms were subsequently evaluated with commercial Ecoplates™ (Biolog, Hayward, CA, USA). Biolog EcoPlate™ contains 31 of the most useful carbon substrates for microbial community analysis (Garland & Mills, 1991), yet do not include e.g., recalcitrant substrates nor specific substrates typical of the soils used in this study (Hunting et al., 2013). Although we cannot directly relate carbon substrate utilization to the actual microbial community and the method does not allow firm conclusiveness on the relative contribution of bacteria and fungi, the number and type of utilized substrates offers a proxy of the functional diversity or resource niche breadth of the microbial community metabolic diversity (Garland, 1999; Elfstrand, Hedlund & Mårtensson, 2007; Rutgers et al., 2016; Krause et al., 2014), in which differences in substrate utilization can indicate that microbial communities can be functionally distinct depending on treatment or sampling area (Hunting et al., 2015; Wang et al., 2015; Zhai et al., 2016; Zhai et al., 2017). Biofilms that developed on the DECOTABs over the course of the experiment were sampled by scraping 1 cm^2^ of the top side of the respective DECOTAB and subsequently dissolved in 50 mL dH_2_O and distributed over the wells. Plates were incubated for 96 h at 18 °C and absorbance was measured at 590 nm using a plate reader.

### Field study: effect of land use on natural microbial resource use and niche breadth

Effects of agricultural practices on microbial resource niche breadth and OM decomposition were studied in an agricultural area in the south-west of the Netherlands (N52.2°; E4.1°), where agricultural drainage ditches are highly interconnected and comparable, and host a diverse macrofaunal community (Ieromina et al., 2014a). The area is intensively used for flower bulb growing (mainly hyacinths, lilies, daffodils and tulips), but also harbors several hectares of grasslands used for dairy farming. Flower bulb growing involves the use and subsequent presence of a wide range of pesticides (including herbicides, fungicides, and insecticides) that are applied continuously in the period February–November. Flowers in the area are grown on sandy soils that are low in organic matter, and soil texture in drainage ditches consists mostly of medium and fine sand, facilitating leaching of high concentrations of pesticides to both groundwater and adjacent surface waters (Ieromina et al., 2014a; Ieromina et al., 2014b). Overall, the ditches in close vicinity of agricultural fields are stagnant and only flow during pumping. The connected waters have comparable physico-chemical conditions and showed to be moderately eutrophic (Ieromina et al., 2016; Hunting et al., 2016); in which nutrient concentration ranges of respectively ${\mathrm{NO}}_{3}^{-}$ and *P* have been observed to be 0.01–0.20 for ditches adjacent to bulb fields; 0.01–0.05 and 0.03 for ditches adjacent to dunes; and 0.01–0.05 and 1.0–2.5 for ditches adjacent to grasslands (Ieromina et al., 2016).

To determine resource niche breadth of microbial communities residing in both the water column and the sediment matrix, microbial communities were sampled from the sediment as well as the water of ditches adjacent to dunes, grasslands and hyacinth bulb fields throughout the research area. Centrifuge flasks (50 mL) were used to sample waterborne microbial communities (six replicates), and distributed over the Ecoplate wells within 2 h after collection. From the same locations, 50 mL of the sediment top layer was carefully sampled to minimize water collection using a 50 mL centrifuge flask (six replicates). In the laboratory, 1 mL of mixed sediment was subsequently sampled with a pipette, diluted 50× with demineralized water and vortexed. Mineral substrate was allowed to settle and the overlying water containing the microbial was subsequently distributed over the Ecoplate wells. Plates were incubated for 96 h at 18 °C and absorbance was measured at 590 nm using a standard plate reader.

DECOTABs were haphazardly distributed over similar ditches within the research area in April 2015. A total number of 12 ditches were selected, four per different ditches adjacent to dunes, grasslands and hyacinth bulb fields, where each ditch received all DECOTAB-treatments. DECOTABs per specific OM treatment were enclosed in a small bag with a mesh size of 500 µm to exclude invertebrate consumption, but allow microbial decomposition (Hunting et al., 2016). DECOTAB decomposition was monitored visually on a weekly basis. Mesh-bags containing DECOTABs were retrieved nine weeks after the start of the experiment. DECOTABs were subsequently rinsed, dried (70 °C for 72 h) and weighed.

### Data analyses

Early assembly of the microbial community in laboratory incubations were analyzed to detect differences in overall differences in substrate utilization via one-way ANOVA with Tukey-HSD pairwise comparisons. Differences in the types of substrate utilized, as measure for microbial functional composition, were tested with a one-way Gower-based ANOSIM (Villéger, Mason & Mouillot, 2008).

Traditional measures of microbial functional diversity that consider the diversity of substrates utilized express functional diversity as metabolic diversity or overall functional diversity, reflecting the average number of substrates utilized and among treatment variation. This approach does not provides insights into within treatment variation in substrate utilization required to assess the breadth of resources utilized. Therefore, resource niche breadth was assessed by determining within treatment Gower-distances considering the square root of nearest roots sensu (Whittaker et al., 2014). Samples to determine resource niche breadth were collected differently in both the water column and the sediment matrix, and therefore possible differences in resource niche breadth under different types of land use were assessed using a one-way NPermanova for both water and sediment. Mass loss of DECOTABs of the different organic matter sources (three levels: Dune, Grass, Bulb) in the different areas (two levels: Grass, Bulb) were analyzed using a two-way ANOVA. Homogeneity of variances of all models was evaluated via Levene’s test for homogeneity. Normality of the model residuals was evaluated via QQ-plots.

## Results

### Laboratory incubation: effect of OM treatment on microbial community assembly

Carbon utilization of the biofilm developed on DECOTABs in our laboratory incubations as visible on the Biolog Ecoplates ranged from 5–11 out of a possible 31 substrates. The overall number of carbon sources utilized did not differ between the different OM treatments (one-way ANOVA: *F* = 1.18, *p* = 0.34), nor did carbon utilization profiles (or overall functional diversity) differ between the different OM treatments (one-way ANOSIM: *R* < 0.01, *p* = 0.42). Bacterial resource niche breadth was also comparable among the different OM treatments (NPermanova, *p* = 0.4).

### Field study: effect of land use on natural microbial functional diversity and functioning

**Figure 1:**
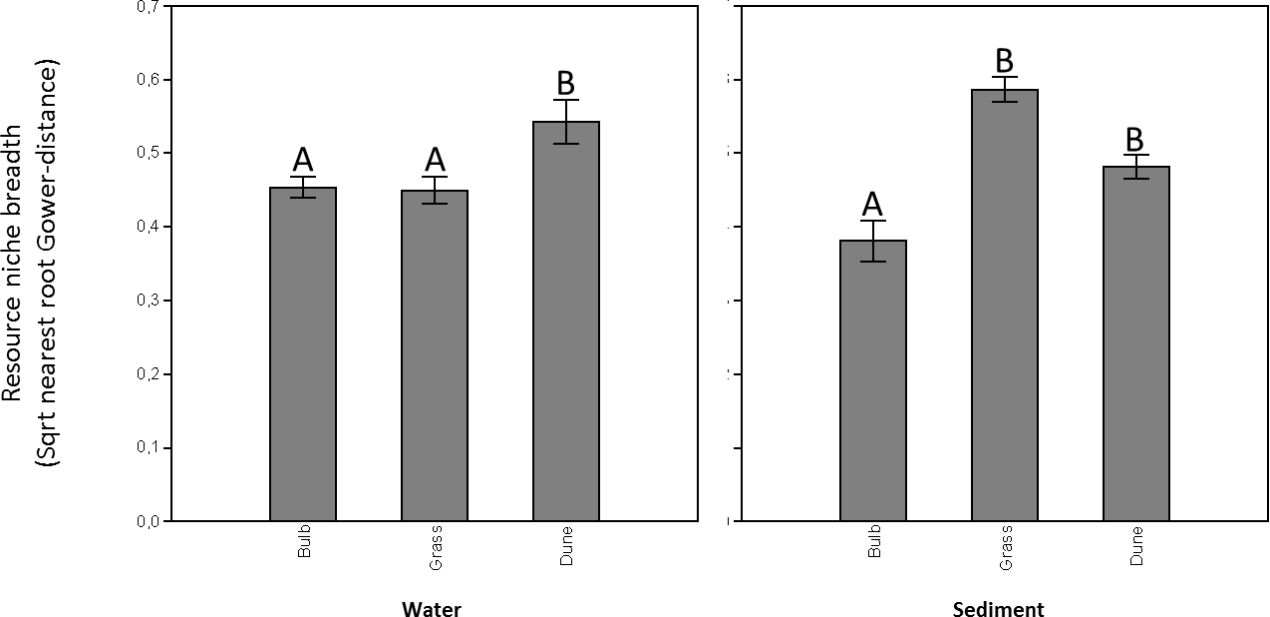
Microbial resource niche breadths (sqrt-Gower based nearest root distances) of microbial communities residing in both the water (A) and the sediment matrix (B) in ditches adjacent to bulb fields, grasslands, and pristine dune areas. Mean (*n* = 6 ± sd) and corresponding letters indicate that microbial resource niche breadths are statistically similar (Gower-based NPermanova, Bonferroni-cerrected pairwise comparison, *p* > 0.05).

Carbon utilization by microorganisms collected from the water in ditches adjacent to the different land uses as visible on the Biolog Ecoplates ranged from 16–24 substrates. The overall number of carbon sources utilized did not differ between the different OM treatments (one-way ANOVA: *F* = 2.81, *p* = 0.10), nor did carbon utilization profiles (or overall functional diversity) differ between the different OM treatments (one-way ANOSIM: *R* = 0.2, *p* = 0.10). Resource niche breadth of the microbial communities collected from the water adjacent to hyacinth bulb fields and grasslands were significantly lower compared to the resource niche breadths of communities collected from dune area ditches (Fig. 1A; NPermanova, Bonferroni-corrected pairwise comparison, *p* = 0.01).

Carbon utilization of microorganisms collected from the sediment in ditches adjacent to the different land uses as visible on the Biolog Ecoplates ranged from 6–23 substrates. The overall number of carbon sources utilized differed between the different OM treatments (one-way ANOVA: *F* = 5.11, *p* = 0.02), in which the number of carbon substrates utilized was significantly lower in microbial communities obtained from sediment adjacent to hyacinth bulb fields compared to those from sediment adjacent to grasslands and dunes (Tukey-HSD, *p* < 0.05). Carbon utilization profiles, a proxy for overall functional diversity, differed between the different OM treatments (one-way ANOSIM: *R* = 0.3, *p* < 0.01), in which carbon utilization profiles were significantly different for microbial communities from sediment adjacent to hyacinth bulb fields compared to those from sediment adjacent to grasslands and dunes (Bonferroni-corrected pairwise comparison *p* < 0.05). A SIMPER analysis did not provide evidence that observed differences were strongly substrate dependent (Supplemental Information 5). Resource niche breadth of the microbial communities residing in the sediment adjacent to hyacinth bulb fields was significantly lower compared to the resource niche breadths of communities collected from the grassland and dune area ditches (Fig. 1B; NPermanova, Bonferroni-corrected pairwise comparison, *p* < 0.05).

**Figure 2:**
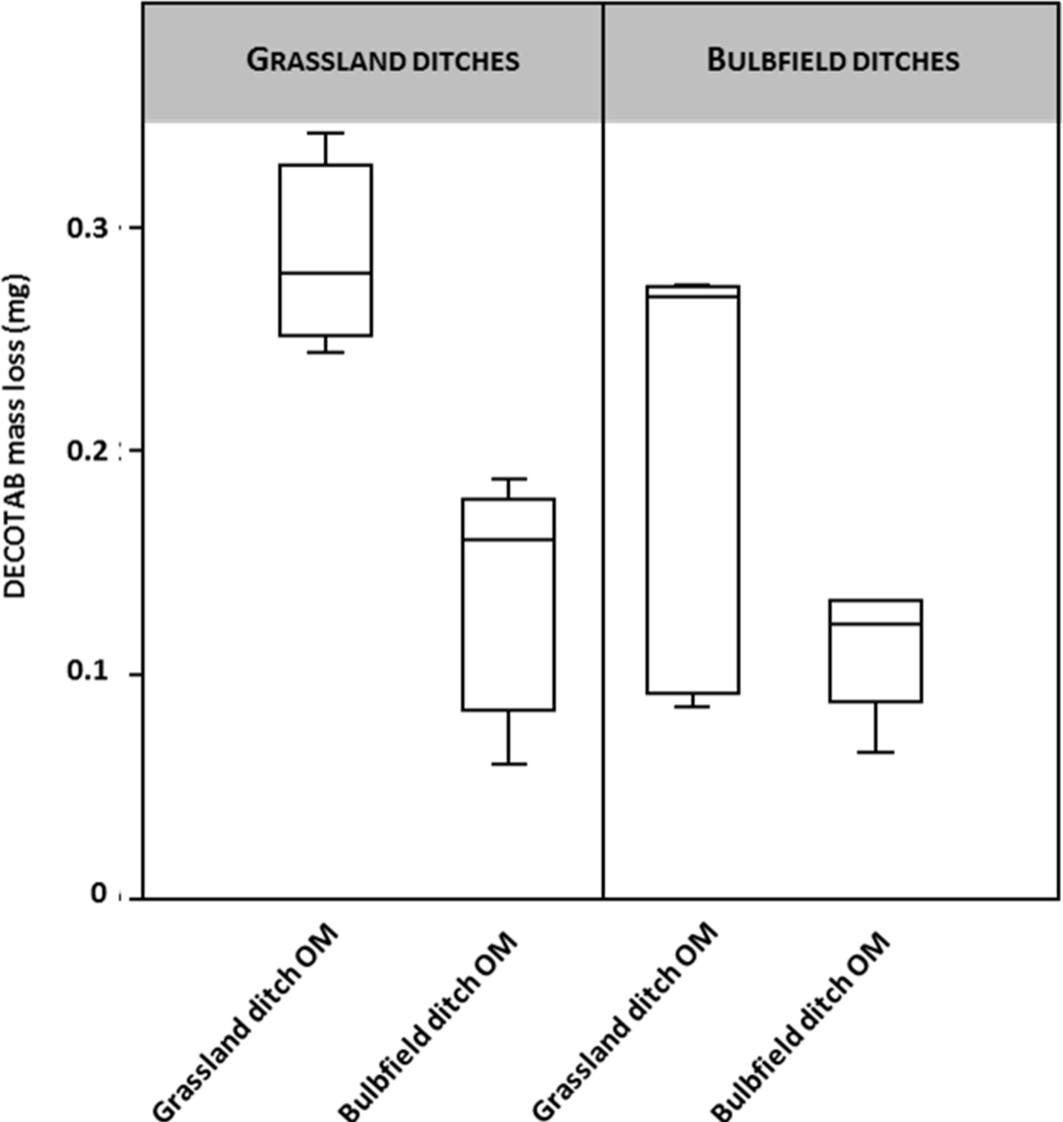
DECOTAB mass loss (mg) of organic matter derived from ditches adjacent to bulb fields and grasslands, incubated in ditches adjacent to both bulb fields and grasslands (*n* = 4). Two-way ANOVA showed significant effects of OM source (grassland ditch OM and bulb field ditch OM), but no effects of ditch type (grassland or bulb field).

DECOTAB mass loss was determined to test for the effects of agricultural practices on OM decomposition (Fig. 2). Ditches in the dune area ran dry over the course of the experiment, resulting in dehydrated DECOTABs. The Dune ditch setting was therefore omitted from further analysis. A two-way ANOVA testing for both OM-source and ditch type suggests a significant effect of OM type (F 9.78; *p* = 0.005), in which decomposition of bulb field derived OM—DECOTABs was significantly retarded in ditches adjacent to grasslands compared to the decomposition of grassland derived OM DECOTABs (Fig. 2A). There was no significant effect of ditch type (grassland and bulb fields, *F* = 2.8; *p* = 0.11). Grassland derived OM DECOTABs appeared to be highly variable in bulb field ditches (Fig. 2B), but no significant interaction effect was observed (F: 3.71; *p* = 0.07).

## Discussion

In this study we observed a clear difference in patterns in niche differentiation between our laboratory incubations and observed in natural ditches. Microbial resource niche breadths were observed to vary in natural ditches depending on adjacent land use. In contrast, in our laboratory incubations, differences in OM composition and history did not result in differences in resource utilization profiles and resource niche breadth during the initial stages of microbial community assembly. It should be noted that resource niche breadths in our laboratory incubations were substantially lower that the resource niche breadths of the natural communities in agricultural drainage ditches. This is likely because microbial community assembly on organic matter from ditches is a slow process that requires longer periods for differences in resource niche breadths to become visible (Fukami et al., 2010). In addition, we used single point measurements in our field sampling where communities likely show temporal variations, and it thus remains uncertain whether our laboratory results reflect the relevant natural conditions and stages of community succession. Most studies to date aiming at assessing the mechanistic underpinnings of diversity effects on ecosystem function have relied on laboratory incubations (Salles et al., 2009; Hunting et al., 2015). Although these studies could provide valuable information on key factors driving microbial community assembly and processes, the data presented here hint that patterns emerging during short term laboratory incubations do not necessarily reflect responses of natural microbial communities in their natural environment.

Within agricultural drainage ditches, microbial resource utilization and niche breadth were observed to vary depending on the type of land use. However, these patterns appeared inconsistent between microbial communities residing in the water column or sediments matrix, suggesting that there is no clear driver affecting overall microbial resource niche breadth (e.g., nutrient loads or direct toxicity of agricultural chemicals). Our research area covered ditches adjacent to a pristine dune area, hyacinths bulb fields and grassland. The OM within these ditches thus represents a complex mixture of natural OM that realistically reflects local agricultural practices, OM inputs and different stages of OM degradation. It remains speculative which mechanisms underlie the observed differences in substrate utilization. Resource composition and availability influence microbial production and community (functional) diversity (Hättenschwiler & Vitousek, 2000; Muylaert et al., 2002; Docherty et al., 2006; Judd, Crump & Kling, 2006; Langenheder & Prosser, 2008; Fonte et al., 2013; Hunting et al., 2013), while resource history of microbial communities and complementarity among specialists can contribute strongly to productivity and carbon turnover (Mou et al., 2008; Strickland et al., 2009; Gravel et al., 2011; Langenheder & Székely, 2011). The interplay between resource diversity and microbial metabolic diversity and community responses to environmental changes thus potentially influences the spatio-temporal properties of ecosystem processes. Results obtained in this study indicate that OM within the ditches exerted a strong bottom up effect on the functional composition of the microbial communities. It can be speculated that the observed differences in resource utilization are driven by the sorption of extensively applied agricultural chemicals to OM. Differences in microbial community resource niche breadths were observed between communities residing in the water column and the sediment matrix. This is likely due to differences in the properties of the agricultural chemicals, in particular solubility, which largely determines whether agricultural chemicals remain in the water or accumulate in the sediment. A similar mechanism has been observed previously considering OM palatability for invertebrates (Hunting et al., 2016). Although only two types of agricultural practices were assessed in this study and the mechanistic underpinnings remain uncertain, our findings suggest that environments that are typically more strongly affected by agricultural practices harbor a narrower niche breadth. This hints that microbial resource niche-differentiation could potentially be used to assess the ecological health and functioning of natural communities, for instance to assess whether environmental impacts of agricultural pesticide applications resonate beyond the boundaries of the treated fields (see e.g., Malaj et al., 2014; Stehle & Schulz, 2015; Vijver et al., 2017).

While adverse effects of anthropogenic pressures have been observed on links between microbial diversity and specific processes such as denitrification (e.g., Powell, Welsh & Hallin, 2015), links between microbial resource niche breadth and measures of overall functioning (Carbon turnover, decomposition) remain uncertain. Here, effects of agricultural practices on resource niche breadths of natural microbial communities were evaluated in conjunction with degradation of natural OM. Our results show that a reduced microbial resource breadth coincides with retarded decomposition of OM collected from ditches adjacent to hyacinth bulb fields, both in bulb field and grassland ditches. However, when OM collected from grassland ditches were offered to microbial communities in ditches adjacent to bulb fields, OM degradation rates were not significantly lower. Since primary production is removed in bulb fields and thereby results in reduced subsidies of (often lower quality) fresh organic material (Didden, 2001), these results seem to indicate that microbial communities in ditches adjacent to bulb field have difficulties degrading the native, low quality OM, yet do have the potential of efficiently degrading higher quality OM. Therefore, our findings seem to suggest that natural microbial communities exhibit high levels of functional redundancy, and that measured microbial community resource niche breadth reflects the assembly on available OM (and the quality thereof) rather than actual impairment of the microbial community. This also implies that, although microorganisms may serve as a sensitive gauge to detect environmental perturbations, the use of commonly used alien OM sources (for instance standardized plant litter used in litter bags) will unlikely accurately detect actual bacterial resource niche differentiation. While it seems conceivable that pressures can affect a specific bacterial clade and inherent process (see e.g., Powell, Welsh & Hallin, 2015), data presented here contributes to the notion that natural microbial communities appear more resilient and functionally redundant when overall measures of ecosystem functioning (i.e., carbon turnover, OM degradation) are considered (e.g., Weedon et al., 2017). In this, it can be speculated that OM quality and local physico-chemical parameters are important in driving a bottom-up control on the actual OM degradation rates observed in the studied agricultural ditches (e.g., Graham et al., 2016).

## Conclusions

Although microorganisms are key to the performance of many ecosystems, they are rarely considered in efforts to monitor or predict the effects of anthropogenic pressures on ecosystem health and functioning. Here we evaluated effects of agricultural land use on the resource niche breadth of microbial communities during initial community assembly and within an environmentally relevant setting, and how these translate to OM degradation. While microbial resource niches were not observed to differ in laboratory incubations, resource utilization and niche breadth of natural microbial communities were observed to be constrained in drainage ditches adjacent to agricultural fields. This outcome coincides with retarded degradation of natural OM collected from ditches adjacent to hyacinth bulb fields, in which microbial communities showed functional redundancy when offered a seemingly higher quality substrate in their natural environment. Although we only focused on a limited number of land use practices, results presented here therefore hint that agricultural practices can impose constraints on microbial functional diversity by reducing the quality OM resources, which can subsequently translate to confined microbial resource niche differentiation and reduced organic matter degradation rates. This study thereby presents a case to consider actual microbial resource utilization and niche differentiation within natural settings to obtain a better understanding of the hazards and risks of land use practices and the effect of environmental perturbations on adjacent aquatic ecosystems.

##  Supplemental Information

10.7717/peerj.4175/supp-1 Supplemental Information 1 Decotab mass loss DECOTAB mass loss (mg) of organic matter derived from ditches adjacent to bulb fields and grasslands, incubated in ditches adjacent to both bulb fields and grasslands (*n* = 4). Two-way ANOVA showed significant effects of OM source (grassland ditch OM and bulb field ditch OM), but no effects of ditch type (grassland or bulb field).

10.7717/peerj.4175/supp-2 Supplemental Information 2 Water ecoplate Microbial resource niche breadths (sqrt-Gower based nearest root distances) of microbial communities residing in both the water (A) and the sediment matrix (B) in ditches adjacent to bulb fields, grasslands, and pristine dune areas Mean (*n* = 6 ± sd) and corresponding letters indicate that microbial resource niche breadths are statistically similar (Gower-based NPermanova, Bonferroni-cerrected pairwise comparison, *p* > 005).

10.7717/peerj.4175/supp-3 Supplemental Information 3 Ecoplate lab ANOSIM text only.

10.7717/peerj.4175/supp-4 Supplemental Information 4 Ecoplate field Microbial resource niche breadths (sqrt-Gower based nearest root distances) of microbial communities residing in both the water (A) and the sediment matrix (B) in ditches adjacent to bulb fields, grasslands, and pristine dune areas Mean (*n* = 6 ± sd) and corresponding letters indicate that microbial resource niche breadths are statistically similar (Gower-based NPermanova, Bonferroni-cerrected pairwise comparison, *p* > 005).

10.7717/peerj.4175/supp-5 Supplemental Information 5 Simper analysis

## Additional Information and Declarations

### Data Availability

The following information was supplied regarding data availability: The raw data has been provided as a Supplemental File.
